# Wayside Detection of Wheel Minor Defects in High-Speed Trains by a Bayesian Blind Source Separation Method

**DOI:** 10.3390/s19183981

**Published:** 2019-09-14

**Authors:** Xiao-Zhou Liu, Chi Xu, Yi-Qing Ni

**Affiliations:** 1 Hong Kong Branch of the National Rail Transit Electrification and Automation Engineering Technology Research Center, Hong Kong, China; xiaozhou.liu@connect.polyu.hk (X.-Z.L.); herbert.xu@connect.polyu.hk (C.X.); 2 Department of Civil and Environmental Engineering, The Hong Kong Polytechnic University, Hung Hom, Kowloon, Hong Kong, China

**Keywords:** wheel minor defect, high-speed train, online wayside detection, Bayesian blind source separation, FBG sensor array

## Abstract

For high-speed trains, out-of-roundness (OOR)/defects on wheel tread with small radius deviation may suffice to give rise to severe damage on both vehicle components and track structure when they run at high speeds. It is thus highly desirable to detect the defects in a timely manner and then conduct wheel re-profiling for the defective wheels. This paper presents a wayside fiber Bragg grating (FBG)-based wheel condition monitoring system which can detect wheel tread defects online during train passage. A defect identification algorithm is developed to identify potential wheel defects with the monitoring data of rail strain response collected by the devised system. In view that minor wheel defects can only generate anomalies with low amplitude compared with the wheel load effect, advanced signal processing methods are needed to extract the defect-sensitive feature from the monitoring data. This paper explores a Bayesian blind source separation (BSS) method to decompose the rail response signal and to obtain the component that contains defect-sensitive features. After that, the potential defects are identified by analyzing anomalies in the time history based on the Chauvenet’s criterion. To verify the proposed defect detection method, a blind test is conducted using a new train equipped with defective wheels. The results show that all the defects are identified and they concur well with offline wheel radius deviation measurement results. Minor defects with a radius deviation of only 0.06 mm are successfully detected.

## 1. Introduction

Wheel out-of-roundness (OOR)/tread defects can impose damage to both rail tracks and vehicle components such as sleepers, wheelsets, and bearings, increasing the likelihood of derailment and undermining operational safety and ride comfort owing to high vibration amplitudes [1,2]. They can also generate ground vibration and noise that annoy residents living around the rail line [3,4,5]. Furthermore, while a wheel may continue to operate if it carries a small flat or polygonal shape, it is subjected to a cyclic impact load every time it rotates and the service life of key components on the vehicle-track system would be reduced [6,7]. For high-speed rail (HSR) and trains, wheel defects are the prime factor leading to faults and failures of both vehicle components and rail infrastructure in service. Due to high running speed, a wheel defect with small radius deviation within the current manufacturing/maintenance tolerance has the potential to give rise to abnormal vibration by exciting various vibration modes for the wheelsets [8,9].

To understand the causes and consequences of wheel defects, a large number of theoretical investigations and experiments have been carried out with the intention to reveal the initiation and development mechanism [2,5,7,10,11,12,13], as well as to perceive their effects on railway operation and safety through dynamic simulation [4,5,9,11,14,15,16,17,18]. In terms of controlling the development of wheel defects, previous studies [5,13] show that the most common and effective strategy is wheel re-profiling. In most cases, wheel defects, if caught in early stages, can be removed or machined out by re-profiling before damage becomes disastrous [19]. However, the existing mileage-based wheel re-profiling may run counter to operator’s expectation by increasing the maintenance cost and reducing the service life of wheelsets. Therefore, there is a large economic incentive for adopting a condition-based maintenance (CBM) scheme which can detect and replace out-of-round wheels in time, to reduce maintenance costs for wheelsets and efficiently preventing the hazards imposed by wheel defects. Wayside wheel condition monitoring is such an efficient method under CBM scheme [20]. With the help of a wheel condition monitoring scheme, the wheelset maintenance activities can then be optimized, thereby allowing whole life costs to be reduced based on a life-cycle cost assessment.

There have been a variety of methods for wayside wheel defect detection. Included are wayside wheel load impact detectors (WILDs) [1,21,22,23,24,25], wayside rail acceleration detectors [26,27], wayside acoustic detectors [28,29], and wayside detectors based on laser and video camera techniques [30,31], etc. Our recent work [32] has given a brief review and comparison of wheel condition monitoring methods. It reveals that online monitoring can be more effective than offline/static inspection for wheel condition assessment and defect detection. Compared with the vehicle-borne wheel defect detection method, the wayside detectors are more suitable for massive wheel inspections. Compared with other online wayside detectors, including laser and video camera based detectors and vibration and acoustic detectors, the strain gauge based detectors confer unique benefits: (i) they are immune to train-induced vibration so they are suitable for in-service train detection, while the performance of laser and video camera based detectors can be limited by vibration during train passage; and (ii) the response signals collected by strain gauges can directly refer to wheel impact, while the effect of the excitation due to the neighboring wheels on the response features is not ignorable when the accelerometer-based and acoustic detectors are employed. The sensors in the impact detection system are usually strain gauge rosettes [33,34] or fiber Bragg grating (FBG) sensors [22]. However, most of the existing WILDs only focus on the amplitude of impact load to decide whether an impact is too great for the vehicle to remain in service [6]. If the maximum load exceeds the preset threshold, an alarm will be given. It is suitable for detection of large defects which often occur when trains run on normal-speed railways, metro lines, and freight lines. The wheel defects they investigated are deep (around 1 mm) or wide (wider than 0.1 m) flats. However, for high-speed trains, as small as 0.5 mm (radius deviation) local defect and 0.04 mm polygonal wear can be critical. As such, a more sophisticated system is needed for minor defect detection. Besides, when trains pass over the instrumented segment at low speeds, the anomalies generated by wheel defects on rail response will not be easily identified.

Therefore, in order to make a rational decision about whether a wheel should be re-profiled, a well-developed data processing procedure is demanded. This paper pursues Bayesian blind source separation (BSS) with Gaussian process (GP) model to extract the defect-sensitive feature. A defect detection procedure is then developed, which enables potential wheel minor defect identification in light of the online-monitored rail response data. The algorithms in the detection procedure are coded in MATLAB environment so that defective wheel(s) can be detected and the defect(s) can be located automatically during the passage of in-service trains.

The rest of this paper is outlined as follows. The wayside wheel condition monitoring system for rail strain data acquisition is introduced in Section 2. Section 3 presents the proposed Bayesian BSS-based wheel defect identification method, and its in-situ verification for high-speed train wheel detection is presented in Section 4. Finally, some conclusions are drawn in Section 5.

## 2. FBG-Based Wayside Wheel Defect Detection System

### 2.1. FBG Sensing System in Wayside Detection

The major challenges of the existing wayside wheel defect detection systems—including various types of WILDs, hot box detectors and laser-based systems, etc.—when applied to HSR, include the clearance required for the equipment to be installed, the need of power supply for sensors and more space claimed by deploying data acquisition system. These problems would be eliminated when using FBG sensors, which offer many advantages over conventional electrical sensors, such as immunity to electro-magnetic interference (EMI), long life-time, remote sensing and self-referencing, compact size, massive multiplexing capability, high reliability and durability, low cost and easy implementation. Specifically, in developing wayside wheel condition monitoring system, the following features of FBG sensing techniques are particularly favorable:

- Assurance of immunity to electromagnetic field: most of the conventional wheel condition monitoring systems, either resistance strain gauge- or accelerometer-based, are vulnerable to EMI induced by high voltage power supply system of modern HSR [23];
- Massive multiplexing capability: HSR always has strict requirements on clearance, which can be problematic for conventional sensing systems when considerable measuring points are needed. In contrast, FBG-based sensing system allows the use of hundreds of sensing points (FBGs) in a single fiber cable. This ability facilitates easy installation on HSR tracks with light-weight trackside equipment;
- High reliability and durability: the FBG-based sensing system can operate for more than 20 years without losses in performance even in extreme climate, such as heavy rains and snows, strong winds, or extremely hot summer days, and corrosion environment and large shocks caused by track maintenance work [22];
- Long conduction distance: the FBG-based sensing system can offer up to 100 km distant detection [23], because the optical fiber has a salient advantage in long-distance transmission with much lower signal attenuation. This allows the monitoring equipment to be installed far away from the instrumented rail section where the sensors are deployed and both the sensors and connecting fibers at the instrumented zone require no power supply.

### 2.2. FBG-Based Wayside Wheel Defect Detector

**Figure 1:**
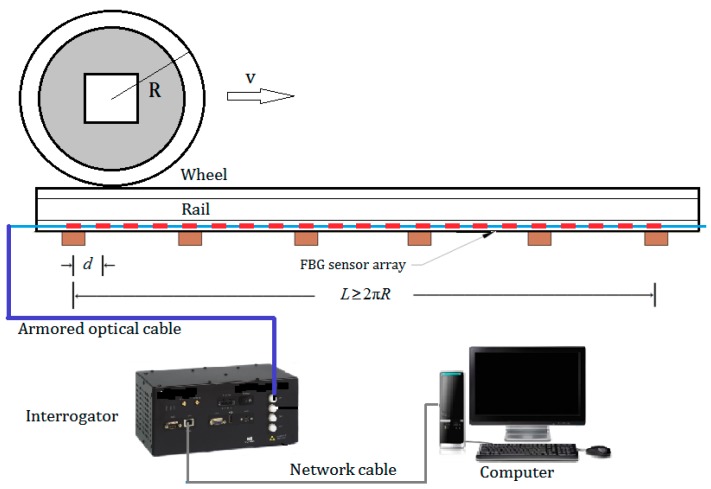
Deployment of FBG sensor array and configuration of the online monitoring system.

An FBG-based wayside rail strain response detector was developed in our recent research [35], where two FBG arrays were devised for deployment on feet of parallel rails (both left and right rails) to capture the features of potential wheel defects. The configuration of FBG array is determined based on numerical simulation presented in [36]. Through this simulation, the rail dynamic strain response subject to the excitation of defective wheel is precisely evaluated, from which the features of localized anomalies caused by wheel local defect can be revealed. This proves the feasibility of mounting strain gauges on rail foot to collect response data containing features of potential defects. The FBG sensor array deployed on rail is thereby designed which, by densely distributing the FBG sensors along a rail segment, can capture with high fidelity the localized anomaly caused by flat-defect if it exists. Figure 1 shows the deployment of FBG sensor array and configuration of the devised system. Each FBG can measure the longitudinal strain of rail foot caused by bending moment of the cross-section under the excitation of wheel dynamic load. The length of the array is slightly longer than the distance rolled over by the wheel for a complete cycle (i.e., the circumference of the wheel tread). The interval of the FBGs (denoted as *d*) along the array is around 0.15 m. This is to ensure that a few neighboring FBG sensors can concurrently detect the defect-sensitive features when a potential defect hits at any location within the instrumented rail section.

Because FBG sensors are used, power supply is not required at the railway site, the interrogator, as data logger can be installed with computer in a control room/office far away from the instrumented rail section with the use of a multi-core armored fiber optic cable to realize data transmission. As shown in Figure 1, the configuration of the devised online monitoring system consists of: (i) two FBG strain gauge arrays installed on the feet of the parallel rails; (ii) a high-speed interrogator for data collection; and (iii) a computer with data acquisition and processing software for system control and data analysis. The online detector collects rail response data at a sampling rate of 5000 Hz. The high sampling rate renders the time interval of sampling much shorter than the predicted time difference of localized anomalies, thereby the desired features can be captured.

## 3. Wheel Defect Identification

With the online detector presented in Section 2, the rail strain responses to the excitation of passing wheels can be collected by FBG sensors deployed on the instrumented section. To detect the defective wheels as well as identify the local defects in an accurate and timely manner, a signal processing method is needed. Besides, as aforementioned, the defect detection for high-speed trains should focus on minor defects with small radius deviation, so the wheel defect detection method needs to be carefully designed in order to extract features from rail response signals that are sensitive to the defects.

The procedure of wheel defect detection in compliance with monitoring data of rail responses can be divided into three steps: firstly, the monitoring data are pre-processed using a signal extraction method, as detailed in Section 3.1. Then, the defect-sensitive feature is extracted by Bayesian BSS, as described in Section 3.2. Lastly, the potential defect is confirmed and identified by a defect confirmation scheme based on the Chauvenet’s criterion, as presented in Section 3.3.

### 3.1. Strain Response Extraction

**Figure 2:**
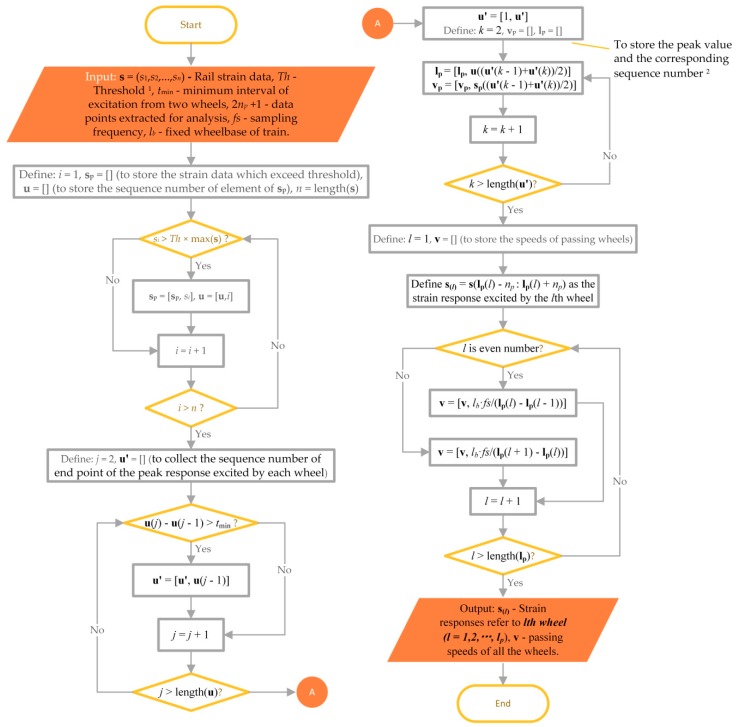
Procedure of rail response extraction. ^1^ The user-defined threshold *Th* is based on the knowledge of passing trains (e.g., heavy wagons, locomotives, metro trains, or high-speed trains). For high-speed trains concerned in this study, for example, the variance of the peak values is relatively small, the value of *Th* can be greater than 0.5. ^2^ In this step, the maximum strain value may not be the peak point considering that the noise in observation data may generate false peaks.

The requirement of real-time wheel condition monitoring and defect detection means a need for signal processing algorithm that can automatically extract response excited by each wheel from whole time history of rail strain response. To this end, the first step is to search the peak values and the corresponding time slots. The number of peaks is equal to the number of passing wheelsets. A signal processing strategy comprising four loops is developed for the response extraction, as shown in Figure 2.

**Figure 3:**
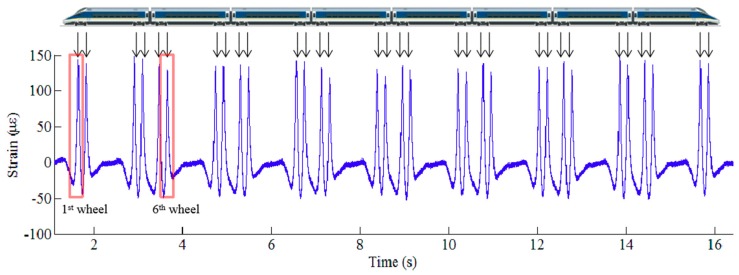
Measured strain response acquired by an FBG sensor deployed at rail foot.

**Figure 4:**
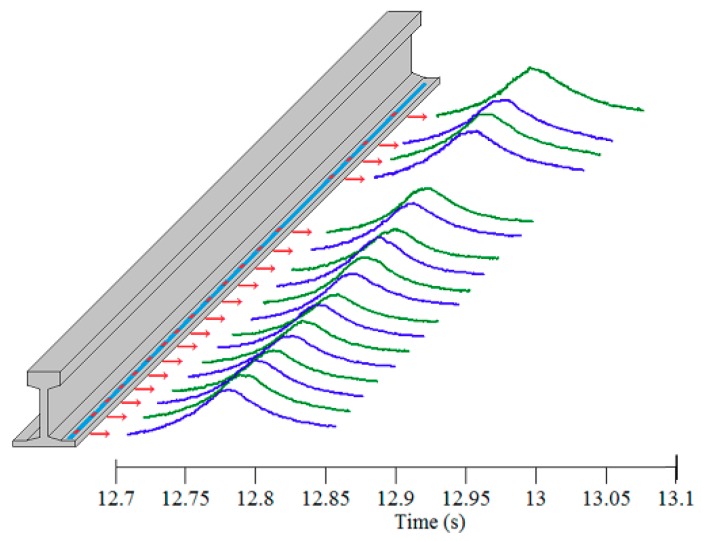
Strain responses acquired by different FBG sensors.

Figure 3 shows the measured strain response acquired by an FBG sensor deployed at rail foot during the passage of an eight-car train. The time history of the strain response exhibits 32 peaks in accordance with 32 wheelsets. By conducting the response extraction procedure, the strain responses corresponding to all the wheels can directly refer to the excitation of passing wheels. As such, we can extract the rail strain responses automatically with the proposed procedure, which offer a window to obtain the section of interest from the waveform of rail strain response, as indicated in Figure 3. The strain responses around their peaks obtained from different FBG sensors when a wheel passes over the instrumented rail section are illustrated in Figure 4.

### 3.2. Defect-Sensitive Feature Extraction Based on Bayesian BSS

The datasets of rail response referring to all passing wheels have been obtained in Section 3.1. As shown in Figure 4, the output strain data contain a major trend that reflects the variation of rail strain response excited by the wheel passage, as well as disturbance caused by both wheel tread roughness and signal noise. In this section, the rail strain response data will be decomposed and the feature corresponding to wheel local defect will be extracted by employing Bayesian BSS, so that the effect of tread roughness of the passing wheel can be quantified.

#### 3.2.1. Bayesian BSS

The aim of BSS is to estimate *n* signals (sources) and a mixing function from the *m* sole observations of mixtures of them. To solve the BSS problem, as an ill-posed inverse problem, the most used prior knowledge is to assume the mutual independence of each series of source signals, which leads to the development of independent component analysis (ICA) methods [37,38] and the second-order blind identification (SOBI) methods [39]. These methods cannot fully consider the temporal structure in the source signals and the difference of noise power among different channels. For rail strain response signals, the time-varying feature can be seen clearly, as shown in Figure 4. Therefore, in signal decomposition, the temporal structure in source signals should be taken into account. Besides, the FBG sensors on the array may have different performances at different locations because of uncertainties generated during the manufacturing and installation. These factors inevitably have some influence on the monitoring data. Thus, different noise power should be assigned to different channels. In recognition of this, the present study uses a Bayesian BSS framework, in which a hierarchical fully Bayesian approach for BSS problem is built.

#### 3.2.2. Assumptions and Model Establishment

The basic form of a BSS problem at time *t* can be written as

(1) X(t)=Y(t)+Z(t)=AS(t)+Z(t)

where **X**(*t*) = [*x*_1_(*t*), *x*_2_(t), ..., *x_m_*(*t*)]*^T^* is a vector of size *m* standing for noisy observations, **S**(*t*) = [*s*_1_(*t*), *s*_2_(t), ..., *s_n_*(*t*)]*^T^* is a vector of size *n* containing the hidden sources mixing in the observation signals; **A** is called ‘mixing matrix’ representing the transfer function from the sources to the sensors; **Z**(*t*) = [*z*_1_(*t*), *z*_2_(t), ..., *z_m_*(*t*)]*^T^* is the noise vector of size *m*; **Y**(*t*) is observation signals without noise contamination. Unlike traditional BSS techniques (e.g., SOBI method) which assume that different noise sequences have a same variance, the present study considers a diagonal covariance matrix Σ*_Z_* to model the noise sequences **Z**(*t*). Thus,

(2) $$p\left(Z|\sum_{Z}\right)=\prod_{t=1}^{L}N\left(Z(t);0,\sum_{Z}\right)$$

where *L* is the length of sequences **X**, **S**, and **Z**; and $N\left(Z(t);0,\sum_{Z}\right)$ represents normal distribution with the mean *μ* and variance *σ*^2^. The diagonal elements in the matrix are equal to different noise power *σ_i_*^2^ of the *i*th observation point (i.e., $\sum_{Z_{ii}}=\sigma_{i}^{2}$). After modeling the noise, the likelihood function of the observation **X** can be expressed as

(3) $$p\left(X|A,S,\Sigma_{Z}\right)=\prod_{t=1}^{L}p\left(X(t)|A,S(t),\sum_{Z}\right)=\prod_{t=1}^{L}N\left(X(t);AS(t),\sum_{Z}\right)$$

Due to the fact that each source signal of rail response is temporally correlated, GP prior is applied in the model as a prior distribution for source signals. For any finite dimensions, there always are a mean vector and a covariation matrix to describe a selected set of variables. In this model, each source signal is assumed to be a stationary GP with zero-mean, squared exponential covariation function. Thus, the source prior can be written as

(4) $$p\left(S|K_{j}\right)=\prod_{j=1}^{n}p\left(S_{j}^{T}\right)=\prod_{j=1}^{n}N\left(S_{j}^{T};0,K_{j}\right)$$

where **S***_j_* = [*s_j_*(*t*_1_), *s_j_*(*t*_2_), ..., *s_j_*(*t_L_*)] is the *j*th source signal; *K_j_* is the covariation matrix with a GP kernel expression of any two times *t* and *t*’

(5) $$K_{j}\left(t,t^{'}\right)=\rho \times \exp\left(-\frac{{\left|t-t^{'}\right|}^{2}}{2h_{j}}\right)$$

where ρ is a scale factor of the kernel that indicates the power of the generated GP, *h_j_* is the hyperparameter of the *j*th source signal.

For the prior of mixing matrix **A**, we consider discriminative inferences for different measuring points (FBGs) in modeling, and it is written as

(6) $$p\left(A|\varepsilon \right)=\prod_{i=1}^{m}\prod_{j=1}^{n}p\left(a_{ij}\right)=\prod_{i=1}^{m}\prod_{j=1}^{n}N\left(a_{ij};0,\varepsilon_{ij}\right)$$

where *a_ij_* is the element in the *i*th row and the *j*th column of the mixing matrix; *ɛ_ij_* is the variance of *a_ij_* and it can be considered as a hyperparameter of the mixing matrix prior.

For the distribution of the hyperparameters of noise (Σ*_Z_*) and mixing matrix (*ɛ*), the conjugate prior for the variance in Gaussian likelihood is used

(7) $$p\left(\sum_{Z}\right)=\prod_{i=1}^{m}p\left(\sum_{Zii}|\alpha_{Z},\beta_{Z}\right)=\prod_{i=1}^{m}IG\left(\sigma_{i}^{2}|\alpha_{Z},\beta_{Z}\right)$$

(8) $$p\left(\varepsilon \right)=\prod_{i=1}^{m}\prod_{j=1}^{n}p\left(\varepsilon_{ij}|\alpha_{a},\beta_{a}\right)=\prod_{i=1}^{m}\prod_{j=1}^{n}IG\left(\varepsilon_{ij}|\alpha_{a},\beta_{a}\right)$$

where *α_Z_, β_Z_, α_a_*, and *β_a_* are known parameters in inverse-gamma distribution.

Due to the non-negativity of the hyperparameter of source (*h*), gamma distribution is used to describe this hyperparameter’s statistical feature

(9) $$p\left(h\right)=\prod_{j=1}^{n}p\left(h_{j}\right)=\prod_{j=1}^{n}G\left(h_{j}|\alpha_{S},\beta_{S}\right)$$

where *α_S_* and *β_S_* are two known parameters in the above gamma distribution.

After modeling of all the prior distributions and introducing distributions of the source and mixing matrix hyperparameters, the joint posterior distribution can be calculated by the Bayes’ theorem, which is expressed as

(10) $$\begin{array}{l} p\left(A,S,\Sigma_{Z},h,\varepsilon |X\right)\propto p\left(X|A,S,\Sigma_{Z}\right)\times p\left(A|\varepsilon \right)\times p\left(\varepsilon \right)\times p\left(h\right)\times p\left(\Sigma_{Z}\right)\\ \;=\prod_{t=1}^{L}N\left(X\left(t\right);AS\left(t\right),\Sigma_{Z}\right)\times \prod_{j=1}^{n}N\left(S_{j}^{T};0,K_{j}\right)\times \prod_{i=1}^{m}\prod_{j=1}^{n}N\left(a_{ij}|0,\varepsilon_{ij}\right)\\ \;\times \prod_{i=1}^{m}\prod_{j=1}^{n}IG\left(\varepsilon_{ij}|\alpha_{a},\beta_{a}\right)\times \prod_{j=1}^{n}G\left(h_{j}|\alpha_{S},\beta_{S}\right)\times \prod_{i=1}^{m}IG\left(\sigma_{i}^{2}|\alpha_{n},\beta_{n}\right) \end{array}$$

To solve this joint posterior, both Gibbs sampling and Metropolis–Hastings (M-H) algorithm, as two MCMC methods, are used in Bayesian BSS model to estimate **A**, **S**, Σ*_Z_*, *h*, and *ɛ*. The procedure, which was detailed in our previous research [40], consists of: (i) generating samples of the source, mixing matrix, noise covariance matrix and mixing matrix hyperparameter from the corresponding conditional posteriors *p*(**S**|**X**,**A**,Σ_Z_,*h*), *p*(**A**|**S**,**X**,Σ_Z_,*h,ɛ*), *p*(Σ_Z_|**A**,**S**,**X**), and *p*(*ɛ*|**A**) by Gibbs sampling; (ii) deriving the expression of these conditional posteriors; and (iii) deriving the posterior of the source hyperparameter *p*(*h*|**S**), which does not belong to a standard conjugate family by the M-H algorithm.

#### 3.2.3. Defect-Sensitive Feature Extraction

**Figure 5:**
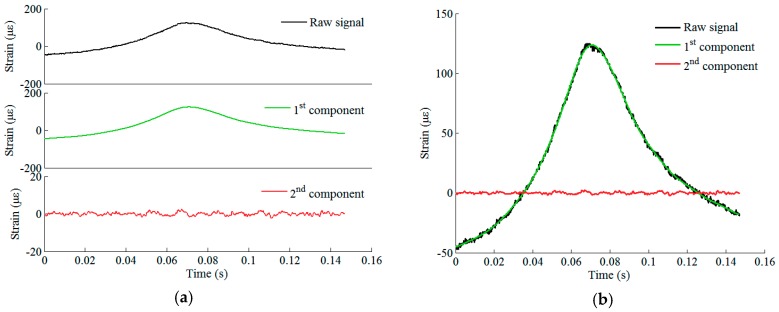
Raw signal of rail response to the excitation of a healthy wheel and its decomposed components: (**a**) plotted in different panels; (**b**) plotted in the same panel.

**Figure 6:**
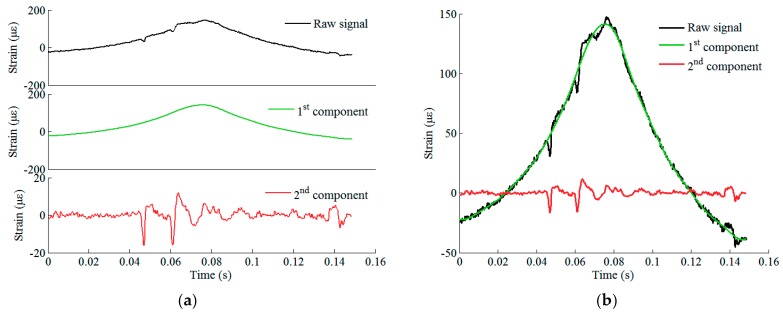
Raw signal of rail response to the excitation of a defective wheel and its decomposed components: (**a**) plotted in different panels; (**b**) plotted in the same panel.

The original strain response acquired by an FBG sensor situated at rail foot under the excitation of an eight-car EMU (32 wheels) is shown in Figure 4. By using the proposed Bayesian BSS method, two sources are derived. The raw response data can thereby be decomposed into two components by multiplying sources by mixing weights. Figure 5 and Figure 6 illustrate two sets of original signals of rail response and their decomposed components. They are generated by a healthy wheel and a wheel with local defects, respectively. It is seen that in both cases, the first component is the trend of the original response signal and it reflects the rail response to an ideally rounded wheel, whereas the second component is the response excited by wheel roughness only. Comparing the two cases, the defect feature can be extracted by analyzing the second component.

### 3.3. Defect Identification

Pursuant to the signal decomposition and feature extraction of rail strain responses described above, this section will identify anomalies in the time history from the second signal component obtained by Bayesian BSS, targeting to detect potential defects. To the end, a threshold is to be set by a criterion for outlier detection. If a number of data points of the decomposed signal exceed the threshold, they will be recognized as localized anomalies.

**Figure 7:**
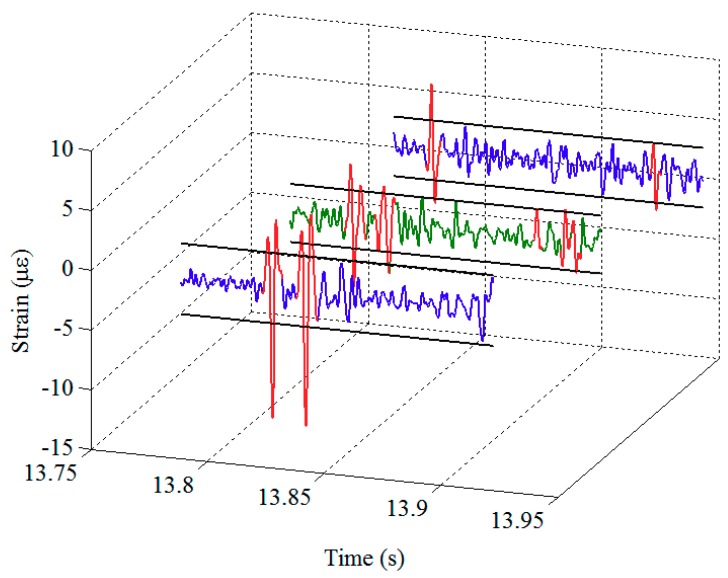
Detection of localized anomalies from the normalized strain data – an example of two strain response datasets: blue and green curves—normalized strain time histories from two different FBGs; black straight lines—the upper and lower thresholds specified by the Chauvenet’s criterion; red curves—the anomalies identified using the Chauvenet’s criterion.

Regarding the choice of criterion, it is considered that track structures and vehicle components can sustain the dynamic loads in all but the worst cases, without catastrophic failure [11]. Similarly, wheel defects that may generate such wheel–rail interaction force should be rare. Therefore, the Chauvenet’s criterion is a suitable method in identifying localized anomalies that are likely to be wheel defects. A threshold (limit) for judging the anomalies from the normalized data can thereby be placed. The upper and lower limits of the probability band given by the Chauvenet’s criterion are expressed in Equation (11) and Equation (12), respectively.

(11) $$x_{u}=F^{-1}\left(1-0.25/N|\mu ,\sigma \right)$$

(12) $$x_{l}=2\mu -F^{-1}\left(1-0.25/N|\mu ,\sigma \right)$$

where *x_u_* and *x_l_* are the upper and lower limits of the probability band, *F*^−1^ is the normal inverse function, and *N* is the sample size. Given the lower and upper limits, the anomalies on the time history of normalized strain data can then be easily detected, as shown in Figure 7. Note that in this study, anomalies are the data points that are beyond lower or upper limits and the adjacent data points within a certain range in time series.

So far, the anomalies on the normalized rail strain data are obtained. However, whether these anomalies are caused by actual wheel defects still needs further investigation. Our previous studies [35,36] revealed that a strong evidence for the presence of a wheel defect is that there are more than one anomaly found on the responses collected by different FBGs and these anomalies occur at the same time period. In view of this, we can further examine the anomalies identified by the Chauvenet’s criterion through a comparison of adjacent FBGs. Specifically, if an anomaly is concurrently identified from the normalized responses collected by different FBGs at the same time period, a wheel local defect can be confirmed. The features of the potential defect, including the relative response amplitude and its location on the wheel tread, can subsequently be obtained. An example of the screening mechanism was given in [35].

The implementation procedures for online wheel defect detection in a timely manner are as follows: when a train passes over the instrumented rail section, the detector is triggered to collect data and the three-step algorithm is employed for instant data processing and evaluation. The acquired data are pre-processed first to obtain the rail response corresponding to the specific wheel load excitation (step I); afterwards, the defect-sensitive feature extraction is conducted through signal decomposition based on Bayesian BSS (step II); lastly, the localized anomalies obtained through feature extraction are used to identify potential wheel local defect by the Chauvenet’s criterion (step III).

In the proposed defect detection process, there are several factors that may have effects on detection results. Among them, three issues need further exploration: the speed variation of passing trains, the temperature effect on strain measurement, and the location of FBGs with respect to sleepers. Their possible influences are discussed in the following:

- The speed variation of passing trains: The process of train passage lasts from seconds to dozens of seconds, so it is possible that the train is speeding up or slowing down during this process and the speed is not constant. However, as described in the proposed method, the condition of wheels is assessed individually, that is, the detection of each wheel is free from the interference of other wheels. Since the instrumented rail section with FBG array is only about 3 m long, the speed of each wheel is unlikely to change dramatically during its passage across the instrumented section. In addition, it has been proven that the dynamic strain monitoring data of rail obtained under different constant running speeds of a train give rise to consistent wheel defect detection results as long as the running speed is instantly measured and enough large dynamic strain of the rail is excited by the passing wheel. It is observed that when the train’s running speed is lower than 20 km/h, the anomaly stemming from minor wheel defect is difficult to perceive in the measured rail dynamic strain response.
- The temperature effect on strain measurement: For strain measurement using FBG sensors, the temperature effect usually should not be ignored, since the output wavelength of FBG sensors can shift with temperature variation. However, temperature-induced wavelength change would not influence the performance of the proposed method. This is because the wavelength change caused by temperature variation mainly results in the change of baseline of the output signal. The influence of temperature can be easily eliminated by deducting the mean value of wavelength before or after train passage. Particularly in the proposed method, after pursuing BSS, the change of temperature will be reflected in the first component (source) rather than the second component (source), the latter being used for wheel defect detection. Also, the temperature variation during the short time of the wheel’s passage across the instrumented section is ignorable.
- Different locations of FBGs with respect to sleepers: In this study, the FBG sensors on the array have different locations with respect to sleepers. These FBGs measure the rail strain due to bending, and the measurement result may be influenced by the distance of the sensor from the sleeper. Therefore, it is necessary to compare the signals generated by different FBGs on the array. As shown in Figure 4, under the excitation of the same wheel, the waveforms of the rail strain responses at different locations are similar. Even if there are slight differences in the amplitude of response peak, this kind of difference is mainly reflected in the first component after signal processing using BSS, rather than in the second component. Therefore, the detection results would not be affected by this issue.

## 4. In-Situ Verification

In this section, the proposed online detector is deployed on a rail line to verify its capability to collect singles and detect multiple wheel local defects through a blind test. Based on the test results, the performance of the devised system in local defect detection will be assessed.

### 4.1. Implementation of Online Detector

**Figure 8:**
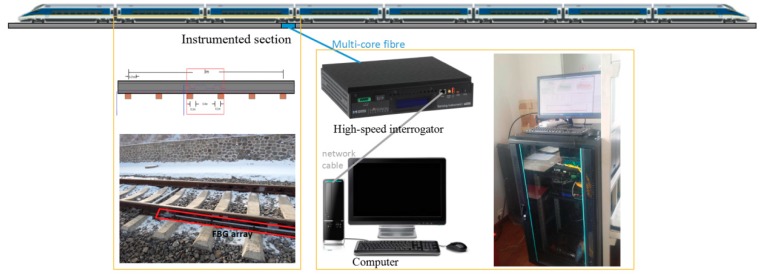
Configuration of the online wheel condition monitoring system.

The devised online wheel defect detector presented in Section 2 has been implemented on a rail line, as shown in Figure 8. The devised system has a trigger module which allows the interrogator to collect wavelength data from the FBG array automatically when there is a train passing over the instrumented rail section. The monitoring data of rail strain response will be stored in a hard disk and sent to the data processing and analysis module, which integrates the wheel defect identification algorithms presented in Section 3. In this way, the condition of the wheel tread and defect information (if any) can be obtained and displayed in real time.

### 4.2. Blind Test

**Figure 9:**
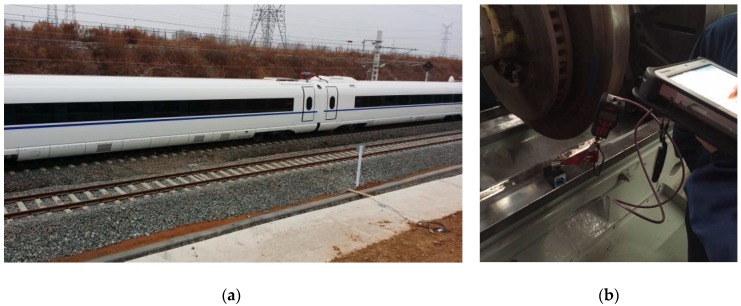
(**a**) The test train of an eight-car high-speed EMU; (**b**) In-depot offline wheel inspection by radius deviation measurement.

To verify the proposed defect detection method, a blind test was conducted by operating a train with potential wheel local defects on the instrumented rail. The test train is a new high-speed EMU equipped with several defective wheels, as shown in Figure 9a. The distance between bogie pivot centers of the test train is 18 m and bogie wheelbase is 2.5 m. The train passes over the instrumented rail several times and the running speed ranges from 20 km/h to 50 km/h. The defective wheels have single or multiple wheel defects on their treads, but the defects were unknown before the test. The proposed defect identification method is applied to process and analyze the monitoring data collected by the online detector. By comparing the online detection results with the results of offline wheel inspection (wheel radius deviation measurement) conducted later in a depot, as shown in Figure 9b, the performance of the detector is evaluated. The detection results and performance analysis of the proposed wheel local defect detection method will be detailed in Section 4.3.

### 4.3. Test Results and Validation

**Figure 10:**
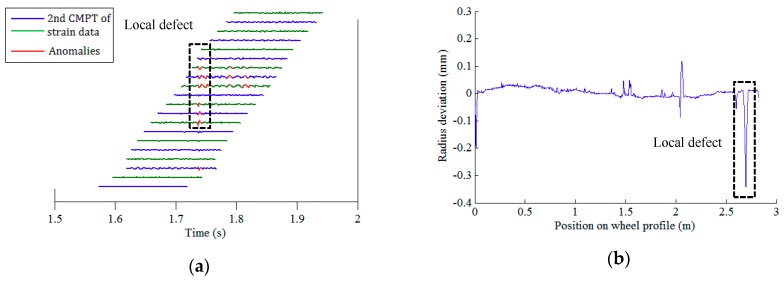
Defect detection results of the right wheel of wheelset no. 1: (**a**) online detection result; (**b**) offline wheel radius deviation measurement.

**Figure 11:**
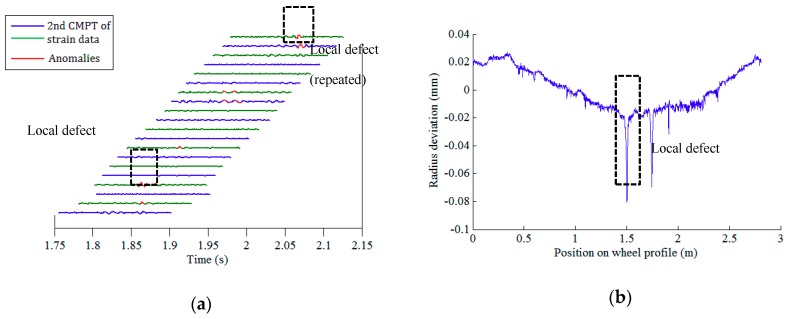
Defect detection results of the right wheel of wheelset no. 6: (**a**) online detection result; (**b**) offline wheel radius deviation measurement.

**Figure 12:**
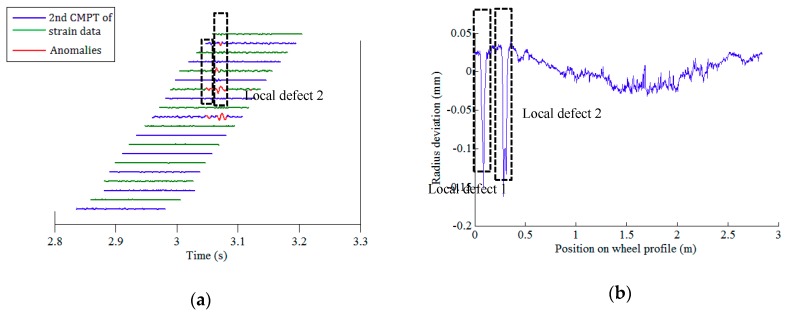
Defect detection results of the right wheel of wheelset no. 24: (**a**) online detection result; (**b**) offline wheel radius deviation measurement.

**Figure 13:**
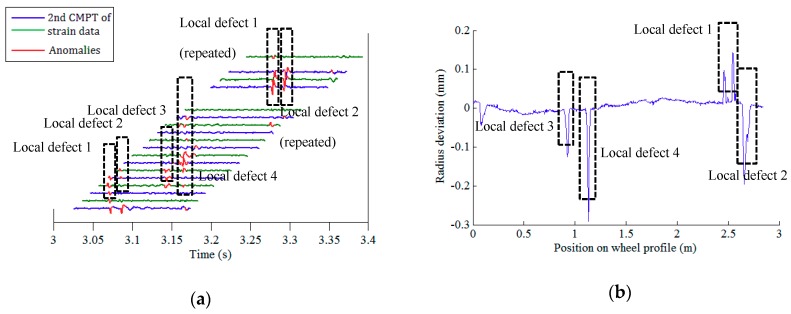
Defect detection results of the left wheel of wheelset no. 27: (**a**) online detection result; (**b**) offline wheel radius deviation measurement.

After conducting data pre-processing (step I) and feature extraction (step II), there are 21 × 64 (21 FBGs on each array, 64 wheels) datasets of the second component of rail response signals. The defect identification algorithm (step III) is then applied to confirm the existence of potential defects. Among the rail strain responses corresponding to all 64 wheels, the right wheels of wheelsets no. 1, 6, and 24, and the left wheel of wheelset no. 27 are identified to have local anomalies. Figure 10a, Figure 11a, Figure 12a and Figure 13a illustrate the second component of raw signals collected by different FBGs on the array (blue and green curves) and localized anomalies (highlighted in red) corresponding to these wheels. It is found that the defect detection results match the radius deviation measurement results (shown in Figure 10b, Figure 11b, Figure 12b and Figure 13b) well in most cases, even in the multiple defect cases. Furthermore, the proposed method has excellent performance in detecting minor defects, whose depth is as low as around 0.06 mm (amplitude is −0.08 mm and baseline is around −0.02 mm), as seen in Figure 11b. It is noteworthy that in multiple defect cases, the signature of a same local defect may occur twice on the response signals collected by the FBG array because it may hit the rail twice and both hits can generate localized anomalies if the contact point is near one end of the FBG array. The online detection result for the left wheel of wheelset no. 27 is such an example, as shown in Figure 13a.

## 5. Conclusions

The demand for ensuring operation safety and reducing maintenance cost for high-speed rail calls for a well-organized CBM scheme which can provide timely and necessary information about the condition of wheels and other key components to the railway operators. To facilitate the application of online wheel defect detection methods, as a critical part of CBM scheme for rolling stocks on HSR, more efforts should be devoted to improving the performance of the existing detection methods, from the perspective of the system configuration design, performance of sensors, data acquisition system, and defect identification algorithms in line with online monitoring data. The work presented in this paper is among these efforts. It is recognized that the FBG-based wayside wheel impact detectors can be more effective for HSR wheel condition assessment and defect detection than conventional detectors. In this study, a new defect identification method for wheel minor defects which are commonly reported to cause abnormal vibration on HSR vehicle-track system is proposed, where an online monitoring system using FBG sensor arrays is employed to collect rail strain responses at multiple locations at rail feet.

In order to automatically detect potential wheel defects, this study proposes a three-step defect identification algorithm to identify local defects in light of the online monitoring data of rail strain responses. The algorithm is carefully designed to reduce both false alarms and missed hits which may induce considerable cost in HSR. Because Bayesian BSS outperforms the conventional BSS techniques in processing signals with temporal structure and quantifying measurement error/uncertainty, the proposed algorithm uses Bayesian BSS to decompose the raw signals and obtain useful features that are sensitive to wheel defects. Through data pre-processing, defect-sensitive feature extraction, and defect confirmation procedure, not only can we identify the defective wheels from all passing wheelsets, we are also able to predict the location of wheel local defects in multiple defect cases.

Blind tests were implemented to verify the performance of the proposed method. Test results indicate that the local defects can be identified with high fidelity, which are in good agreement with the offline measurements of wheel radius deviation taken in a depot. It is found that wheel defects with depth (radius deviation) as low as 0.06 mm can be successfully detected by the proposed method.
